# Individual Differences in the Accuracy of Judgments of Learning Are Related to the Gray Matter Volume and Functional Connectivity of the Left Mid-Insula

**DOI:** 10.3389/fnhum.2017.00399

**Published:** 2017-08-02

**Authors:** Xiao Hu, Zhaomin Liu, Wen Chen, Jun Zheng, Ningxin Su, Wenjing Wang, Chongde Lin, Liang Luo

**Affiliations:** ^1^Collaborative Innovation Center of Assessment Toward Basic Education Quality, Beijing Normal University Beijing, China; ^2^State Key Laboratory of Cognitive Neuroscience and Learning, Beijing Normal University Beijing, China; ^3^School of Sociology, China University of Political Science and Law Beijing, China; ^4^Institute of Developmental Psychology, Beijing Normal University Beijing, China

**Keywords:** functional connectivity, gray matter volume, insula, judgments of learning, metamemory

## Abstract

The judgment of learning (JOL) is an important form of prospective metamemory judgment, and the biological basis of the JOL process is an important topic in metamemory research. Although previous task-related functional magnetic resonance imaging (MRI) studies have examined the brain regions underlying the JOL process, the neural correlates of individual differences in JOL accuracy require further investigation. This study used structural and resting-state functional MRI to investigate whether individual differences in JOL accuracy are related to the gray matter (GM) volume and functional connectivity of the bilateral insula and medial Brodmann area (BA) 11, which are assumed to be related to JOL accuracy. We found that individual differences in JOL accuracy were related to the GM volume of the left mid-insula and to the functional connectivity between the left mid-insula and various other regions, including the left superior parietal lobule/precuneus, bilateral inferior parietal lobule/intraparietal sulcus, right frontal pole and left parahippocampal gyrus/fusiform gyrus/cerebellum. Further analyses indicated that the functional connectivity related to individual differences in JOL accuracy could be divided into two factors and might support information integration and selective attention processes underlying accurate JOLs. In addition, individual differences in JOL accuracy were not related to the GM volume or functional connectivity of the medial BA 11. Our findings provide novel evidence for the role of the left mid-insula and its functional connectivity in the JOL process.

## Introduction

Metamemory refers to the processes of monitoring and controlling memory activities (Nelson and Narens, 1990). One important form of metamemory is prospective metamemory judgment, which refers to prediction of future memory performance (Dunlosky and Metcalfe, 2009). The accuracy of prospective metamemory judgments is typically evaluated by examining the extent to which prospective metamemory judgments discriminate between remembered and forgotten items (Dunlosky and Metcalfe, 2009). If the subjective level of prospective metamemory judgments is higher for remembered items than forgotten items, then the accuracy of prospective metamemory judgments is high. In contrast, prospective metamemory accuracy is low if people give higher judgments to forgotten items. Prospective metamemory judgments can be made either at the stage of acquiring knowledge (called judgment of learning, or JOL) or at the time of retrieval (called feeling of knowing, or FOK) (Nelson and Narens, 1990). JOL is an important type of prospective metamemory judgment, and previous studies have indicated that JOL accuracy is essential for appropriate guidance of subsequent learning and memory processes (Dunlosky and Metcalfe, 2009; Bjork et al., 2013). People with more accurate JOLs can use strategies that are more appropriate in subsequent learning processes, such as allocating study time more appropriately during self-regulated learning or choosing more important and valuable items for restudy (Nelson and Narens, 1990; Dunlosky and Metcalfe, 2009). Given the importance of JOLs in learning processes, the biological basis of the JOL process is an important topic in metamemory research (Schwartz and Bacon, 2008; Chua et al., 2014).

Some lesion-based studies have investigated whether frontal lobe lesions significantly affect JOL accuracy (Vilkki et al., 1998, 1999). For example, Vilkki et al. (1998) asked healthy participants and patients with brain lesions to learn a list of words. Before the recall test, all participants had to make a JOL to predict how many words they could recall in the test. They found that patients with left or right frontal lobe lesions had significantly lower JOL accuracy than the control group. In Vilkki et al. (1999), patients and healthy control participants were required to learn the locations of different faces and make a JOL before the test. The results showed that patients with damaged right frontal lobes showed decreased JOL accuracy. These studies suggest that the frontal lobe may play an important role in the JOL process. However, Vilkki et al. (1998, 1999) did not identify which part of the frontal lobe is essential to JOL accuracy.

In addition to lesion-based studies, functional magnetic resonance imaging (MRI) studies have investigated the brain regions underlying the JOL process (Kao et al., 2005; Do Lam et al., 2012; Yang et al., 2015). An early study by Kao et al. (2005) found that the left lateral frontal cortex, left ventromedial prefrontal cortex and several temporal, parietal, occipital and limbic regions were associated with predicted encoding success (i.e., higher activation for high-JOL trials than for low-JOL trials). They also examined the relationship between individual differences in JOL accuracy and the activation of the regions related to predicted encoding success, and they found that greater JOL accuracy was correlated with higher activation in the ventromedial prefrontal cortex. Another study by Do Lam et al. (2012) separated JOL trials from initial encoding and indicated that the medial prefrontal cortex, medial orbitofrontal cortex and anterior cingulate cortex were more active during high-JOL trials. In addition, Yang et al. (2015) compared the neural activities for encoding trials subsequently given high and low JOLs, and found that the dorsolateral, rostrolateral and ventromedial prefrontal cortex, posterior cingulate cortex, middle temporal gyrus, superior lateral occipital cortex and angular gyrus showed subsequent JOL effects (i.e., higher activation for trials subsequently given high JOLs than those given low JOLs). They also found that greater JOL accuracy was correlated with smaller subsequent JOL effects in the ventromedial prefrontal cortex. In these previous studies, the only region consistently involved in the JOL process was the ventromedial prefrontal cortex, particularly the medial part of Brodmann area (BA) 11. Similarly, a study of FOK has indicated that the activation of medial BA 11 is also related to accurate FOK judgments (Schnyer et al., 2005), suggesting that medial BA 11 is essential to different types of prospective metamemory judgments.

Another important method for investigating the neural correlates of metamemory processes is to examine the relationship between individual differences in brain structure and metamemory accuracy. To our knowledge, no study has examined the relationship between JOL accuracy and brain structure. However, researchers have used structural MRI to investigate the relationship between the FOK process and the gray matter (GM) volume in the brain (Cosentino et al., 2015; Le Berre et al., 2016). For example, Le Berre et al. (2016) asked alcoholic patients to make FOK judgments in the recall phase about whether they could recognize the target word in a subsequent recognition test; they found that greater FOK accuracy was correlated with higher mean GM volume of the bilateral insula. Similarly, Cosentino et al. (2015) indicated that higher mean GM volume of the right insula was related to higher FOK accuracy in older adults. Both studies have shown that individual differences in FOK accuracy are related to the GM volume of the insula.

Although previous studies have investigated the neural correlates of the JOL process from different perspectives, two important issues require further examination. First, although the activation of medial BA 11 has been shown to be consistently associated with the JOL process (Kao et al., 2005; Do Lam et al., 2012; Yang et al., 2015), no study has examined whether individual differences in JOL accuracy are related to the GM volume of medial BA 11. Second, previous structural MRI studies have not investigated whether JOL is also related to the GM volume of the insula, as found in structural MRI studies concerning FOK (Cosentino et al., 2015; Le Berre et al., 2016). Researchers have suggested that in FOK processes, the insula may be related to the monitoring of task performance (Cosentino et al., 2015) or self-inferential processes to generate accurate future estimations (Le Berre et al., 2016). These cognitive processes are also involved in the JOL process (Koriat, 1997; Dunlosky and Metcalfe, 2009). Thus, it is possible that the GM volume of the insula may also be related to JOL accuracy. Investigating these issues could further reveal the neural correlates of the JOL process. To address these issues, in the present study, we first investigated the relationship between JOL accuracy and GM volume in the brain.

In addition, previous neuroimaging studies have mainly focused on the particular brain regions related to JOL process and have not examined whether JOLs are correlated with the functional connectivity between different regions. According to the widely accepted cue-utilization theory, JOLs are inferential in nature and are related to the processing and integration of various cues (e.g., study time, processing fluency) relevant to study materials. When making JOLs, people first monitor and process the cues that may contribute to the memory process and then integrate these cues to generate estimations for future memory performance (Koriat, 1997; Dunlosky and Metcalfe, 2009). Thus, it is possible that JOLs are associated with the coupling between different regions that are related to the processing and integration of different cues. In addition, a previous study suggests that retrospective confidence judgment (RCJ), another type of metamemory judgment made after a memory test, is related to functional connectivity in the brain (Baird et al., 2013). For example, Baird et al. (2013) indicated that individual differences in the accuracy of retrospective confidence in a memory test were significantly correlated with the functional connectivity between the anterior prefrontal cortex, in which the brain structure is related to the accuracy of RCJs (Fleming et al., 2010), and several parietal regions during the resting state. Based on the results concerning RCJs, we speculated that JOLs might also be related to functional connectivity between different regions. Furthermore, making JOLs is similar to the decision-making process (Schwarz, 2004), which is also related to the integration of different information (Bettman et al., 1998; Lim et al., 2013). A previous study has suggested that the evaluation process in decision making is associated with the functional connectivity between several regions of the temporal cortex (which are related to the processing of different information) and the prefrontal cortex (which is related to the integration of information) (Lim et al., 2013). Thus, JOLs may also be associated with the functional connectivity between various brain regions. The second purpose of the present study was to investigate the relationship between JOL accuracy and functional connectivity in the brain.

Taken together, the main goals of the present study were (1) to examine the relationship between individual differences in JOL accuracy and the GM volume in the brain and (2) to investigate the functional connectivity associated with individual differences in JOL accuracy. To achieve the first goal, we analyzed the correlation between individual differences in JOL accuracy and the GM volume. In particular, we examined the correlation between JOL accuracy and the GM volume of medial BA 11 and the insula. Medial BA 11 is consistently involved in the JOL process (Kao et al., 2005; Do Lam et al., 2012; Yang et al., 2015) and has been correlated with JOL accuracy in previous task-related fMRI studies (although the direction of the correlation is controversial; see Kao et al., 2005; Yang et al., 2015). Thus, the GM volume of medial BA 11 may also be correlated with JOL accuracy. In addition, FOK studies have consistently revealed positive correlations between FOK accuracy and the GM volume of the insula (Cosentino et al., 2015; Le Berre et al., 2016). According to the discussion above, some cognitive processes that underlie FOK and may be related to the insula are also involved in the JOL process (Koriat, 1997; Dunlosky and Metcalfe, 2009), and it is possible that the GM volume of the insula might also be related to JOL accuracy. We hypothesized that JOL accuracy might show positive correlation with the GM volume of the insula (similar to FOK studies) and might also show significant correlation with the GM volume of medial BA 11 (although the direction of the correlation is unknown). There are two commonly used methods to analyze the relationship between GM volume and individuals’ performance: the traditional region-of-interest (ROI) approach used in previous FOK studies (Cosentino et al., 2015; Le Berre et al., 2016) and voxel-based morphometry (VBM) analysis. In the ROI-based approach, the correlations between individuals’ performance and the mean GM volume within the ROIs are analyzed. By contrast, VBM analysis permits the calculation of the correlation between individuals’ performance and GM volume voxel by voxel (Luders et al., 2004; Bhaskar et al., 2013). One pitfall of the ROI-based approach is that it is used primarily on large areas, and differences in GM volume in small parts of the ROIs may therefore be overlooked. By contrast, VBM analysis can examine focal differences in brain anatomy and reveal specific clusters related to individuals’ performance (Bhaskar et al., 2013). Thus, in this study, we chose VBM analysis to evaluate the relationship between GM volume and JOL accuracy. We conducted VBM analysis in a mask containing the bilateral insula and medial BA 11 to examine whether JOL accuracy was significantly correlated with the GM volume in these regions.

To achieve the second goal, we used resting-state fMRI to examine whether individual differences in JOL accuracy are related to the resting-state functional connectivity between different brain regions. Resting-state functional connectivity is indexed by correlations in low-frequency fluctuations of the resting-state fMRI signal (Lee et al., 2013). In contrast to task-related brain activity, which is evoked by specific task stimuli, resting-state functional connectivity may characterize the intrinsic functional organization of the brain (Fox and Raichle, 2007) and has high reliability and reproducibility (Lee et al., 2013). Although resting-state functional connectivity is not directly related to any cognitive task, a previous study suggests that individual differences in resting-state functional connectivity can predict task-induced brain activity (Mennes et al., 2010). In addition, resting-state functional connectivity has been shown to be closely related to individual differences in various cognitive functions, such as language, decision making and intelligence (Song et al., 2008; Li et al., 2013; Xiao et al., 2016). In the resting-state analysis, we used the significant cluster found in VBM analysis as a seed region and conducted whole-brain seed-based functional connectivity analysis. Using the cluster found in VBM analysis as the seed region is a widely accepted method in resting-state analysis (Loh and Kanai, 2014; Evans et al., 2015). We hypothesized that if the VBM analysis revealed that the clusters in medial BA 11 or insula were significantly correlated with JOL accuracy, the functional connectivity between these clusters and other regions might also support the process of making accurate JOLs. In addition, to further explore the relationship between JOL accuracy and functional connectivity in the brain, we conducted an exploratory ROI-wise analysis to examine whether JOL accuracy was correlated with the functional connectivity between regions connected to the seed region in the seed-based analysis. We also performed exploratory factor analysis on the functional connectivity found in the analyses above. Factor analysis is a statistical technique that can determine which variables form coherent subsets (factors) that are somewhat independent. Factors are hypothesized to reflect latent constructs or underlying processes that result in correlations between variables (Fabrigar et al., 1999). Thus, factor analysis may provide a better understanding of the functions of the resting-state network related to JOL accuracy by revealing the relationships between functional connectivity outcomes (Jacobs et al., 2014).

## Materials and Methods

### Participants

Thirty-five students (13 males, 22 females; aged 19–28 years) participated in this study. The participants were right-handed, had normal or corrected-to-normal vision, and reported no personal or family history of neurological or psychiatric disorders. This study was carried out in accordance with the recommendations of the Institutional Review Board of the State Key Laboratory of Cognitive Neuroscience and Learning, Beijing Normal University with written informed consent from all subjects. All subjects gave written informed consent in accordance with the Declaration of Helsinki.

### Materials

The materials consisted of 300 two-character Chinese words from the Chinese word database of Cai and Brysbaert (2010). The word frequency varied between 0.24 and 46.29 per million words. The 300 words were randomly divided into two sets (each containing 150 words). The two sets of words did not differ in word frequency, concreteness, familiarity or the number of strokes (*p* > 0.05). One set of words was presented during the encoding phase, and the other set was used as new words during the recognition phase. The assignment of the two sets to the encoding and recognition phases was counterbalanced across participants.

### Task and Procedure

The participants completed two experimental sessions: a behavioral session that included a memory task and an MRI session in which T1-weighted structural images and resting-state fMRI images were acquired. The MRI data were acquired before the behavioral experiment.

The memory task consisted of two phases, encoding and recognition, with 24 h between the two. On the first day, the participants completed the encoding phase. Before beginning the encoding phase, the participants were informed that there would be a recognition test after 24 h in which their memory of the presented words would be tested by asking that they select the previously presented word from two choices (as described below). During the encoding phase, the participants sequentially viewed 150 words that were presented in the center of a screen, with each word presented for 2 s. The words were presented in a random order. Immediately following the presentation of each word, the participants were instructed to rate the probability that they would correctly choose that word during the recognition test. Ratings were made using a 6-point Likert-type scale ranging from 1 (very unlikely) to 6 (very likely) and were entered with the number pad of a keyboard.

The participants left the lab after finishing the encoding phase. They then returned 24 h later to participate in the recognition test, which used a two-alternative forced choice (2AFC) design and contained 150 trials. In each recognition trial, two words were presented on the left and right of the screen. One of these words had been presented in the encoding phase (“old”), while the other word had not been presented previously (“new”). The participants had to press a key to indicate which word was old and then rate their confidence in the accuracy of their response on a 6-point scale ranging from 1 (very low confidence) to 6 (very high confidence). The old and new words were presented in random order, and the location (left or right) of the old words was also randomized.

**FIGURE 1:**
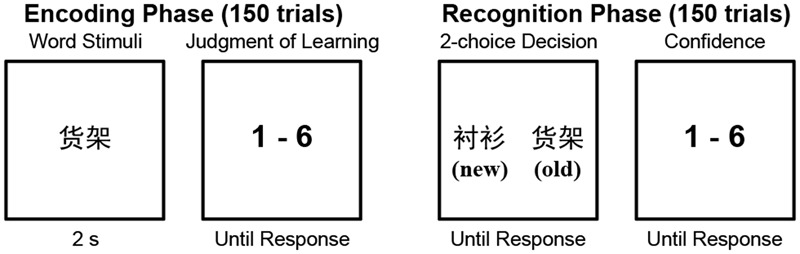
The experimental paradigm of the memory task. The old/new labels were not presented in the recognition phase. They are presented here to explain the experimental procedure.

The memory task procedure is shown in **Figure 1**. Stimulus presentation and response collection were controlled with E-Prime software (Schneider et al., 2002). The confidence ratings made during the recognition phase were not analyzed in this study.

### Quantification of Memory Performance and JOL Accuracy

We used the proportion of correctly answered trials in the recognition test to quantify the participants’ memory performance. JOL accuracy was quantified by calculating the area under the receiver operating characteristic curve (AUROC) (Beaman et al., 2014; Ryals et al., 2016). Previous studies have mainly used two measures of JOL accuracy: Goodman–Kruskal’s gamma and the AUROC. As a traditional measure of JOL accuracy, gamma has been used in behavioral studies and some neuroimaging studies (Nelson, 1984; Kao et al., 2005). However, gamma is susceptible to the tendency to make higher or lower judgment ratings (response bias), which can lead to erroneous interpretations of JOL accuracy (Masson and Rotello, 2009). By contrast, as a non-parametric measure based on Type II signal detection theory (SDT), the AUROC is not influenced by response bias and is a more accurate measure of JOL accuracy than gamma (Ryals et al., 2016). In addition, the AUROC is also used to measure the accuracy of other forms of metamemory, such as RCJs (Ryals et al., 2016; Valk et al., 2016).

The AUROC was determined according to previously published methods (Fleming et al., 2010; Fleming and Lau, 2014) by calculating the area under the Type II receiver operating characteristic (ROC) curve using non-parametric methods. We constructed the ROC curve by treating each JOL level as a criterion that separated high JOLs from low JOLs. For example, we started with a liberal criterion that assigned low JOL = 1 and high JOL = 2–6, then a higher criterion that assigned low JOL = 1–2 and high JOL = 3–6, and so on. For each split of the data, the hit rate *h_i_* = p (high JOL | correct) and false alarm rate *f*_i_ = p (high JOL | incorrect) were calculated and used to construct an x-y point on the ROC curve. The ROC curves were anchored at [0,0] and [1,1]. According to Fleming et al. (2010), an ROC curve that bows sharply upward indicates that the probability of being correct increases rapidly with confidence. Conversely, a flat ROC function indicates a weak link between confidence and accuracy. Application of the Kolmogorov–Smirnov test revealed that the AUROC was normally distributed (*p* > 0.2).

To explore how well the Type II SDT model accounted for the JOL rating data, we fit the following linear regression model (Fleming et al., 2010):

$$\text{z}(\text{h})\;=\;\beta_{0}\;+\;\beta_{1}\text{z}(\text{f})\;+\;\varepsilon$$

where z is the inverse of the cumulative normal distribution function. This model provided an excellent fit to the data (mean *R*^2^ = 0.97).

Some researchers have noted that the AUROC may be confounded by task performance (Galvin et al., 2003). Thus, in the subsequent structural MRI and resting-state fMRI analyses, recognition accuracy was included as a covariate (Valk et al., 2016).

### Structural MRI

#### Data Acquisition

The structural MRI data were acquired using a 3T Siemens Tim Trio MRI scanner at the Imaging Center for Brain Research, Beijing Normal University. Using a magnetization prepared rapid gradient echo (MPRAGE) sequence, high-resolution T1-weighted structural images (TR = 2530 ms, TE = 3.39 ms, inversion time = 1100 ms, flip angle = 7°, 144 sagittal slices, FOV = 192 mm × 256 mm × 256 mm, voxel size = 1.33 mm × 1 mm × 1 mm) were acquired.

#### Voxel-Based Morphometry (VBM) Analysis

Structural brain images were analyzed with the VBM8 toolbox^1^, which was incorporated into SPM8 software^2^ running on MATLAB R2010b (MathWorks, Natick, MA, United States). Preprocessing was completed using the default settings of VBM8. In brief, the following steps were performed: (1) intrasubject bias correction; (2) segmentation into different tissue classes; (3) linear and non-linear registration to the Montreal Neurological Institute (MNI) space (resliced to 1.5 mm × 1.5 mm × 1.5 mm); and (4) modulation of tissue segments using non-linear normalization parameters to account for individual differences in brain size. The normalized GM segments were then smoothed using an 8-mm full-width at half maximum (FWHM) Gaussian kernel.

Statistical analysis was performed using SPM8. We used a mask that contained the bilateral insula and medial BA 11 as the mask in the statistical analysis to examine whether the GM volume in these regions was related to individual differences in JOL accuracy. The bilateral insula region was derived from the xjView toolbox^3^. The BA 11 region was also created from the xjView toolbox, and we used the medial part (-20 < × < 20) of BA 11 in the following analysis (Hogeveen et al., 2016). The participants’ age, gender, recognition accuracy and total volume of brain tissue were included as covariates. Voxels with GM values of <0.2 (absolute threshold masking) were excluded from the analysis to avoid possible edge effects between different tissue types. We applied a height threshold of *p <* 0.001 and a cluster-level threshold of *p <* 0.05 using both the family-wise error (FWE) correction and the non-stationary cluster correction implemented in SPM8 to account for the non-isotropic smoothness of the VBM data (Hayasaka et al., 2004). In addition, we conducted a whole-brain VBM analysis using the same threshold to examine whether JOL accuracy was correlated with the GM volume of other regions in the brain.

We also examined whether the GM volume of the region identified in the analysis described above was significantly correlated with the participants’ recognition performance. The mean GM volume of the region identified in the VBM analysis was extracted, and we calculated the correlation between the GM volume of the region and the participants’ recognition accuracy.

### Resting-State fMRI

#### Data Acquisition

The resting-state fMRI data were acquired using a 3T Siemens Tim Trio MRI scanner at the Imaging Center for Brain Research, Beijing Normal University. Resting-state functional MRI images were obtained using an echo-planar sequence sensitive to blood oxygenation level-dependent contrast (TR = 2000 ms, TE = 30 ms, flip angle = 90°, 33 axial slices acquired interleaved with a 0.7-mm gap, voxel size = 3.125 mm × 3.125 mm × 4.2 mm, FOV = 200 mm × 200 mm × 138.6mm, 200 volumes). The participants were instructed to stay awake and to keep their eyes closed during the functional runs.

#### Data Preprocessing

Preprocessing of the resting-state fMRI data was performed using the DPARSF toolbox^4^ (Yan and Zang, 2010). The first 10 volumes were removed to account for the T1 equilibrium effect, leaving 190 volumes for the final analysis. The functional images were sinc interpolated in time to correct for the slice time differences. Then, the images were realigned with a rigid body linear transformation to correct for head movements. Next, the functional images were co-registered with the corresponding T1 volume and warped into MNI space at a resolution of 3 mm × 3 mm × 3 mm using Diffeomorphic Anatomical Registration Through Exponentiated Lie (DARTEL) algebra in SPM8 (Ashburner, 2007). The images were then spatially smoothed using a 6-mm FWHM Gaussian filter to reduce noise, and the nuisance covariates were regressed out. The nuisance covariates included 24 motion parameters that were calculated from the six original motion parameters using Volterra expansion (Friston et al., 1996). These parameters have been shown to be better at decreasing motion effects than the six original parameters alone (Yan et al., 2013). The white matter and cerebrospinal fluid signals were also covaried out, and linear detrending was performed. The global signal was not included as a nuisance covariate because recent work suggests that regression of the global signal may reduce the accuracy of the connectivity estimates (Saad et al., 2012). The images were then filtered at 0.01–0.1 Hz.

#### Seed-Based Functional Connectivity Analysis

In the VBM analysis, we found that individual differences in JOL accuracy were related to GM volume in a cluster of the left mid-insula (see the Results section). Thus, the first seed region for the seed-based functional connectivity analysis was the left mid-insula identified in the VBM analysis. Although we did not find a relationship between GM volume in medial BA 11 and JOL accuracy (see the Results section), we used the medial BA 11 region defined in the VBM analysis as the second seed region to investigate whether JOL accuracy was correlated with the functional connectivity of medial BA 11. The mean time course of all voxels in each seed region was used to calculate the voxel-wise linear correlations (Pearson’s r) throughout the whole brain. The connectivity maps were transformed from r to z values using Fisher’s z transformation and then submitted to second-level analyses in SPM8. These analyses examined whether the functional connectivity of each seed region was related to the participants’ JOL accuracy. For the connectivity map of each seed region, we calculated the correlation between participants’ JOL accuracy and the *z*-transformed correlation value of each voxel. The participants’ age, gender and recognition accuracy were included as covariates. Similar to previous study (Baird et al., 2013), in the whole-brain analysis, we applied a height threshold of *p <* 0.005 and a cluster-level threshold of *p <* 0.05 with false discovery rate (FDR) correction. In addition to the whole-brain analysis, we also examined whether JOL accuracy was related to the functional connectivity between the left mid-insula and medial BA 11. We conducted a seed-based functional connectivity analysis in the medial BA 11 mask when using the left mid-insula as the seed region and in the left mid-insula mask when using medial BA 11 as the seed region.

We also examined whether the functional connectivity found in the analysis described above was significantly correlated with the participants’ recognition performance. The mean *z*-transformed correlation value for each functional connectivity identified in the analysis above was extracted, and we calculated the correlation between the mean *z*-transformed correlation values of each functional connectivity and the participants’ recognition accuracy. In addition, we investigated whether the relationship between JOL accuracy and functional connectivity found in the analysis above was solely due to the difference in the GM volume of the seed region.

#### ROI-Wise Functional Connectivity Analysis

In the whole-brain seed-based functional connectivity analysis, we found that JOL accuracy was significantly related to the functional connectivity between the left mid-insula and five other regions (see the Results section). To further explore whether individual differences in JOL accuracy were correlated with the functional connectivity between the regions connected to the left mid-insula, we conducted an exploratory ROI-wise analysis using the five regions discovered in the seed-based analysis as ROIs. For each participant, we extracted the mean time series by averaging across all voxels in each ROI and then computed bivariate correlation coefficients for each pair of ROIs. The resultant ROI-to-ROI correlation values were Fisher *z*-transformed. We calculated the partial correlation between JOL accuracy and the z-transformed correlation of each ROI-to-ROI pair, controlling for age, gender and recognition accuracy. To correct for multiple comparisons, we applied a threshold of FDR-corrected *p* < 0.05 for the partial correlation analyses (Benjamini and Hochberg, 1995). We also examined whether the ROI-to-ROI functional connectivity that was related to JOL accuracy was significantly correlated with the participants’ recognition performance.

#### Exploratory Factor Analysis

The seed-based analysis and ROI-wise analysis revealed a brain network with functional connectivity of nine ROI-to-ROI pairs related to JOL accuracy (see the Results section). To examine whether the functional connectivity found in the analyses above could form different subsets, we conducted an exploratory factor analysis (Jacobs et al., 2014). For each participant, we extracted the *z*-transformed correlation of each ROI-to-ROI pair that was significantly correlated with JOL accuracy. The number of factors retained was determined using principal component analysis as an extraction method and a threshold of eigenvalue >1, followed by oblique rotation (Promax). We then conducted a three-step linear regression analysis to investigate whether each of the factors could predict the participants’ JOL accuracy.

## Results

### Behavioral Results

On average, the participants’ recognition accuracy (the proportion of correctly answered trials during the recognition test) was 0.823 (*SD* = 0.092, range 0.59–0.96), which was significantly higher than the chance level (0.5), *t*(34) = 20.7, *p* < 0.001, Cohen’s *d* = 3.50. We then calculated the AUROC of the JOL ratings for each participant. The mean AUROC for all participants was 0.551 (*SD* = 0.073, range 0.371–0.716), which was also significantly higher than the chance level (0.5), *t*(34) = 4.13, *p* < 0.001, Cohen’s *d* = 0.70. These results indicate that participants could significantly predict their future memory performance, although their JOL accuracy was relatively low. In addition, there was no significant correlation between recognition accuracy and the AUROC, *r* (35) = -0.010, *p* = 0.957, suggesting that JOL accuracy may be independent of memory performance. To investigate whether the AUROC was actually independent of the response bias in the JOL ratings, we calculated the Type II bias for each participant according to the method of a previous study (Kornbrot, 2006). The results showed that the AUROC and Type II bias were not significantly correlated, *r*(35) = -0.038, *p* = 0.829, and thus confirmed that the AUROC was not influenced by response bias. In addition, we calculated the correlation between the AUROC and the head motion measurement (mean framewise displacement) in the resting-state fMRI data (Power et al., 2012) and found that this correlation was not significant, *r*(35) = -0.120, *p* = 0.491.

### VBM Analysis Results

**FIGURE 2:**
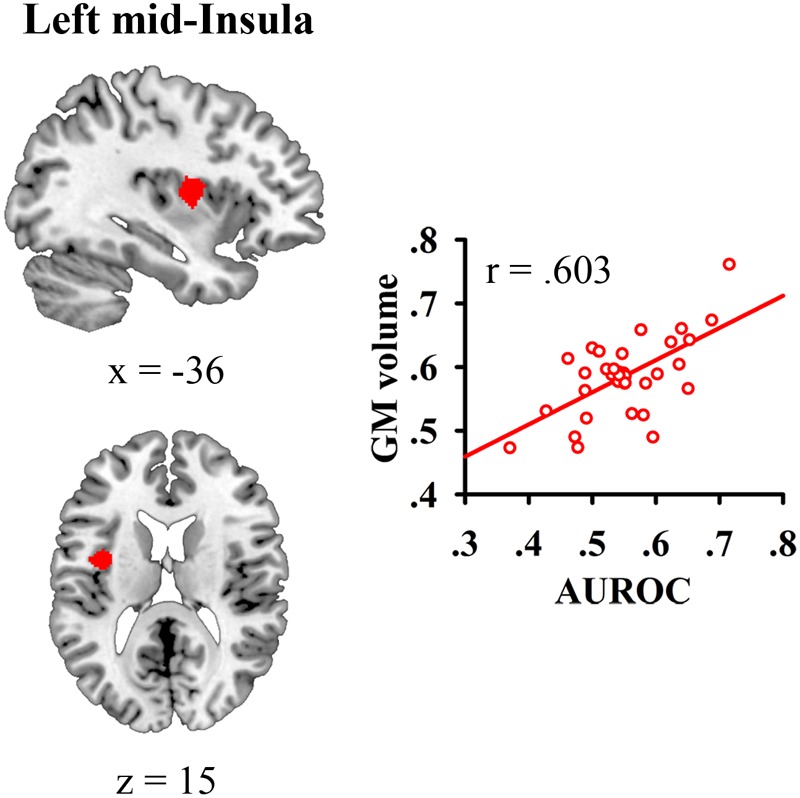
Gray matter (GM) volume of the left mid-insula was related to individual differences in judgment of learning (JOL) accuracy.

We correlated JOL accuracy with the GM volume of each voxel in the mask containing the bilateral insula and medial BA 11 and found that greater JOL accuracy was only correlated with higher GM volume of a cluster in the left mid-insula (BA 13; peak MNI: -40.5, 0, 15; see **Figure 2** and **Table 1**). The volume of this left mid-insula cluster (1006 mm^3^) accounted for 4% of the volume in the left insula (24960 mm^3^) and for 1.3% of the volume in the whole mask (74944 mm^3^). JOL accuracy did not show a negative correlation with the GM volume of the insula or medial BA 11. In addition, whole-brain analysis did not reveal any region in which the GM volume was significantly correlated with JOL accuracy. We also examined whether the GM volume of the left mid-insula was significantly correlated with the participants’ recognition performance and found that this correlation was not significant (*p* > 0.9).

**Table 1:** Brain areas positively correlated with judgment of learning (JOL) accuracy in voxel-based morphometry (VBM) and whole-brain seed-based functional connectivity analyses.

Anatomical areas	BA	Volume (mm^3^)	MNI coordinates			*t-*value
			*X*	*Y*	*Z*	
**VBM analysis**						
L. mid-insula	13	1006	-40.5	0	15	4.31
**Seed-based functional connectivity analysis**						
L. superior parietal lobule	7	3240	-12	-66	63	5.01
L. Precuneus	7		-18	-48	57	4.77
L. parahippocampal gyrus	36	2241	-27	-21	-30	4.33
L. fusiform gyrus	20		-48	-27	-27	4.11
L. cerebellum			-30	-33	-36	4.11
L. inferior parietal lobule/intraparietal sulcus	40	5670	-45	-36	42	4.25
R. frontal pole	10	2619	39	51	21	4.09
R. inferior parietal lobule/intraparietal sulcus	40	2214	45	-39	51	3.75

*L, left; R, right; BA, Brodmann’s area.*

### Seed-Based Functional Connectivity Analysis Results

**FIGURE 3:**
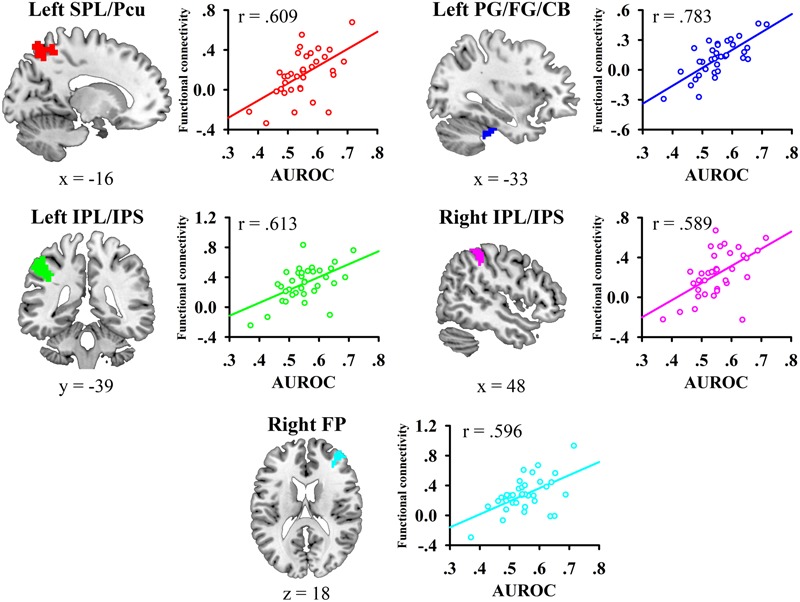
Whole-brain functional connectivity of the left mid-insula predicted individual differences in JOL accuracy. SPL, superior parietal lobule; Pcu, precuneus; IPL, inferior parietal lobule; IPS, intraparietal sulcus; PG, parahippocampal gyrus; FG, fusiform gyrus; CB, cerebellum.

We then conducted whole-brain functional connectivity analysis to investigate whether the functional connectivity of the left mid-insula, which was identified in the VBM analysis, and medial BA 11 was related to the participants’ JOL accuracy. As shown in **Table 1** and **Figure 3**, greater JOL accuracy was significantly correlated with greater functional connectivity between the left mid-insula and five clusters: the left superior parietal lobule/precuneus (SPL/Pcu, BA 7; peak MNI: -12, -66, 63), left inferior parietal lobule/intraparietal sulcus (IPL/IPS, BA 40; peak MNI: -45, -36, 42), right IPL/IPS (BA 40; peak MNI: 45, -39, 51), right frontal pole (BA 10; peak MNI: 39, 51, 21), and a cluster (peak MNI: -27, -21, -30) including part of the left parahippocampal gyrus (BA 36), left fusiform gyrus (BA 20), and left cerebellum. JOL accuracy did not show a significant negative correlation with the functional connectivity between the left mid-insula and any region. In addition, JOL accuracy was not significantly correlated with the functional connectivity between medial BA 11 and any region. To examine whether JOL accuracy was related to the functional connectivity between the left mid-insula and medial BA 11, we conducted seed-based functional connectivity analysis in the medial BA 11 mask when using the left mid-insula as the seed region and in the left mid-insula mask when using medial BA 11 as the seed region. The results revealed that JOL accuracy did not show a significant correlation with the functional connectivity between the left mid-insula and medial BA 11.

We also examined whether the functional connectivity found in the analysis described above was significantly correlated with the participants’ recognition performance and found that no functional connectivity was significantly correlated with recognition accuracy (all *p* > 0.3).

One possibility is that the correlation between JOL accuracy and functional connectivity might be due to differences in the GM volume of the seed region. Thus, we conducted a two-step linear regression analysis to examine whether the relationship between JOL accuracy and the functional connectivity of the left mid-insula was solely due to the correlation between JOL accuracy and the GM volume of the left mid-insula. JOL accuracy was the outcome variable in the regression analysis. In the first step, four control variables (the participants’ age, gender, recognition accuracy, and GM volume of the left mid-insula) were entered in the model. In the second step, the z-transformed correlation values for the functional connectivity between the left mid-insula and the five clusters were entered in the model. The results revealed that the z-transformed correlation values for the functional connectivity could explain the additional unique variance of JOL accuracy [*F_change_* (5,25) = 7.892, *p* < 0.001], which suggested that the functional connectivity of the left mid-insula could predict JOL accuracy independently even when controlling for the GM volume of the left mid-insula.

### ROI-Wise Functional Connectivity Analysis Results

**FIGURE 4:**
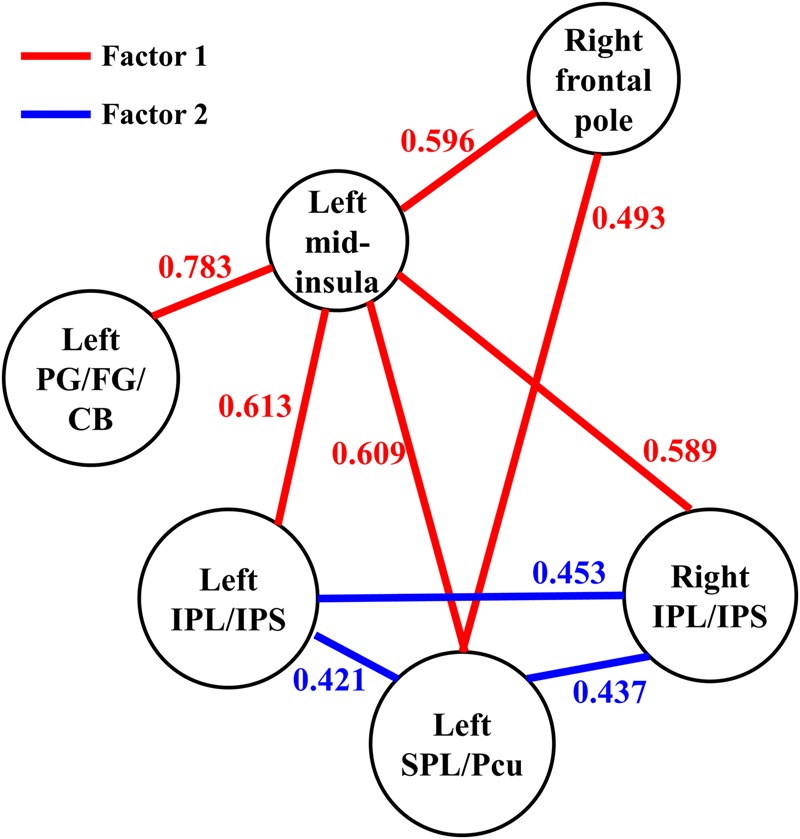
The functional connectivity between the six regions observed in the present study. The parameter for each functional connectivity represents the correlation between the functional connectivity and the participants’ JOL accuracy. SPL, superior parietal lobule; Pcu, precuneus; IPL, inferior parietal lobule; IPS, intraparietal sulcus; PG, parahippocampal gyrus; FG, fusiform gyrus; CB, cerebellum.

We then conducted an exploratory ROI-wise analysis using the five regions found in the seed-based analysis as ROIs to explore whether individual differences in JOL accuracy were correlated with the functional connectivity between the regions connected to the left mid-insula. JOL accuracy showed significant positive correlations with the *z*-transformed correlation values of four ROI-to-ROI pairs (see **Figure 4**): left SPL/Pcu – right frontal pole (*r* = 0.493, FDR-corrected *p* = 0.041), left SPL/Pcu – right IPL/IPS (*r* = 0.437, FDR-corrected *p* = 0.041), left SPL/Pcu – left IPL/IPS (*r* = 0.421, FDR-corrected *p* = 0.041), and left IPL/IPS – right IPL/IPS (*r* = 0.453, FDR-corrected *p* = 0.041). In addition, none of the ROI-to-ROI functional connectivity was significantly correlated with recognition accuracy (all *p* > 0.3).

### Exploratory Factor Analysis Results

We conducted an exploratory factor analysis on the functional connectivity of nine ROI-to-ROI pairs related to JOL accuracy (five found in the seed-based analysis and four found in the ROI-wise analysis) to examine whether the functional connectivity found in the analyses above could form different subsets. The results from the exploratory factor analysis revealed that two factors with an eigenvalue over 1 were extracted, accounting for 66.507% of the variance. Factor 1 had higher loadings on the functional connectivity of six ROI-to-ROI pairs: left mid-insula - right frontal pole, left mid-insula - left SPL/Pcu, left mid-insula – right IPL/IPS, left mid-insula – left IPL/IPS, left mid-insula – left parahippocampal gyrus/fusiform gyrus/cerebellum, and left SPL/Pcu – right frontal pole. Factor 2 had higher loadings on the functional connectivity of three ROI-to-ROI pairs: left SPL/Pcu – right IPL/IPS, left SPL/Pcu – left IPL/IPS, and left IPL/IPS – right IPL/IPS (see **Table 2** and **Figure 4**). In addition, the functional connectivity between the left and right IPL/IPS had factor loadings higher than 0.3 on both factors. We also found that the correlation between the scores of the two factors was significant, *r*(35) = 0.462, *p* = 0.005.

**Table 2:** Factor loadings for each of the functional connectivity related to JOL accuracy (Loadings < 0.30 suppressed).

Functional connectivity	Factor	
	1	2
L. mid-insula – R. frontal pole	0.916	
L. mid-insula – L. SPL/Pcu	0.834	
L. mid-insula – R. IPL/IPS	0.915	
L. mid-insula – L. IPL/IPS	0.952	
L. mid-insula – L. PG/FG/CB	0.620	
L. SPL/Pcu – R. frontal pole	0.533	
L. IPL/IPS – R. IPL/IPS	0.324	0.461
L. SPL/Pcu – L. IPL/IPS		0.871
L. SPL/Pcu – R. IPL/IPS		0.870

*L, left; R, right; SPL, superior parietal lobule; Pcu, precuneus; IPL, inferior parietal lobule; IPS, intraparietal sulcus; PG, parahippocampal gyrus; FG, fusiform gyrus; CB, cerebellum.*

To examine whether these two factors could independently predict individual differences in JOL accuracy, we conducted a three-step linear regression analysis with JOL accuracy as the outcome variable. In the first step, the participants’ age, gender and recognition accuracy were entered in the model as control variables. These variables could not significantly predict JOL accuracy, *R*^2^ = 0.044, adjusted *R*^2^ = -0.049, *F*(3,31) = 0.473, *p* = 0.703. In the second and third steps, the scores of Factors 1 and 2, respectively, were entered in the model. The results revealed that the scores of both factors could explain additional unique variance of JOL accuracy [Factor 1: *F_change_* (1,30) = 35.966, *p* < 0.001; Factor 2: *F_change_* (1,29) = 4.382, *p* = 0.045], suggesting that both factors could predict JOL accuracy independently while controlling for age, gender and recognition accuracy [final regression model: *R*^2^ = 0.622, adjusted *R*^2^ = 0.557; *F*(5,29) = 9.553, *p* < 0.001; age *B* = -0.012, 95% CI [-0.021, -0.003]; gender *B* = 0.026, 95% CI [-0.012, 0.065]; recognition accuracy *B* = -0.088, 95% CI [-0.283, 0.108]; Factor 1 *B* = 0.046, 95% CI [0.026, 0.066]; Factor 2 *B* = 0.021, 95% CI [0, 0.042]].

## Discussion

The present study investigated the neural correlates of individual differences in JOL accuracy. Participants completed a memory task in which they made JOLs about their recognition performance, and we used structural MRI and resting-state fMRI to examine whether individual differences in JOL accuracy were correlated with the GM volume and the resting-state functional connectivity in the brain. We found that the GM volume of the left mid-insula could predict individual differences in the participants’ JOL accuracy. The participants’ JOL accuracy was also correlated with the resting-state functional connectivity between the left mid-insula and various other brain regions, including the left SPL/Pcu, bilateral IPL/IPS, right frontal pole and left parahippocampal gyrus/fusiform gyrus/cerebellum, and the functional connectivity related to JOL accuracy could be divided into two subsets. Our findings provide novel evidence for the involvement of the left mid-insula and other distributed brain regions and their functional connectivity in the processes related to accurate JOLs and suggest that these regions may play an important role in the JOL process.

Our study found that greater JOL accuracy was related to higher GM volume of the left mid-insula. Our results are consistent with previous FOK studies indicating that greater FOK accuracy is correlated with higher GM volume of the insula (Cosentino et al., 2015; Le Berre et al., 2016). These studies suggest that in FOK processes, the insula may be related to performance monitoring (Cosentino et al., 2015) or to self-inferential processes for generating accurate future estimations (Le Berre et al., 2016). JOL and FOK are both prospective metamemory judgments and involve similar cognitive processes (Dunlosky and Metcalfe, 2009). Thus, the insula may play similar roles in FOK and JOL. In the JOL process, the insula may be associated with the monitoring of the performance in the memory-encoding phase and the generation of the predictions for future memory test. In contrast to previous FOK studies that used ROI-based approach to analyze the GM volume of the insula (Cosentino et al., 2015; Le Berre et al., 2016), this study used VBM analysis and found that the GM volume of the left mid-insula was specifically correlated with JOL accuracy. Previous studies have indicated that the mid-insula plays an important role in the evaluation of bodily state (i.e., interoception) (Craig, 2011). In the interoceptive process, the mid-insula re-represents the primary neural representations of the state of the body and integrates the representations of the body with other neural inputs to form a combined representation of the individual’s internal and external environment (Craig, 2009, 2011). Thus, it is possible that in the JOL process, the left mid-insula may monitor and process the neural representations of different information or cues related to the performance in the memory-encoding phase and then integrate the cues to generate estimations for future memory test (Koriat, 1997; Kao et al., 2005). In addition, Chua et al. (2009) showed that metamemory process was associated with less activity in brain regions related to the processing of external visual stimuli, and with greater activity in regions responsible for internally directed cognition. Chua et al. (2009) suggest that metamemory is characterized by both a shift toward internally directed cognition and away from externally directed cognition. One possibility is that the left mid-insula may serve as a hub between external representation and internal representation and may be related to the shift toward internally directed representation. Moreover, Critchley et al. (2004) suggest that in the interoceptive process, the insula is related to the subjective feeling states that may underlie the conscious representation of our internal bodily processes. Perhaps the left mid-insula is also associated with the conscious representation of our experience in the memory-encoding phase. Future research should further examine the role of the left mid-insula in the JOL process.

One difference between this study and previous FOK studies is that previous studies have indicated that FOK accuracy is related to the right or bilateral insula (Cosentino et al., 2015; Le Berre et al., 2016), whereas this study revealed a significant relationship between JOL accuracy and the left mid-insula. One possible explanation is that the insula in different hemispheres might be related to prospective metamemory judgments at different stages: JOL is made in the encoding phase, while FOK is made during the memory test (Dunlosky and Metcalfe, 2009). In addition, we also note that results from previous FOK studies are not identical (Cosentino et al., 2015; Le Berre et al., 2016), and it is possible that the different tasks used in this study and previous studies may lead to differences in lateralization. For example, Cosentino et al. (2015) asked participants to learn information about different people and make FOK judgments. They found that FOK accuracy was related to the right insular volume. By contrast, Le Berre et al. (2016) required participants to learn unrelated word pairs and found that the FOK accuracy was related to the GM volume of the bilateral insula. In contrast to previous studies, this study required participants to learn a list of words and perform a recognition test. Future research should use VBM analysis to examine the relationship between the GM volume in the insula and both types of prospective metamemory judgments in a single experiment.

Contrary to our hypothesis, individual differences in JOL accuracy were not related to the GM volume of medial BA 11, which is activated in the JOL process according to previous task-related fMRI studies (Kao et al., 2005; Do Lam et al., 2012; Yang et al., 2015). In addition, our results from resting-state fMRI analysis showed that the functional connectivity of medial BA 11 was not correlated with JOL accuracy. Structural MRI and resting-state fMRI analyses aim to reveal the variability in brain volume or resting-state activity in relevant neural structures that reflect individual differences in performance. It does not strictly follow that the identified region is more active during the relevant tasks, because the relationship between task-related fMRI findings and the results of structural MRI and resting-state fMRI analyses is complex (Mennes et al., 2010; Kanai and Rees, 2011; McCurdy et al., 2013). Future research should combine structural MRI, resting-state fMRI and task-related fMRI to further investigate the neural correlates of the JOL process. In addition, even in previous task-related fMRI studies concerning JOL, the direction of correlation between JOL accuracy and the activation of medial BA 11 has been controversial (Kao et al., 2005; Yang et al., 2015). For example, Kao et al. (2005) required participants to learn pictures of scenes and make JOLs for all pictures. They found that JOL accuracy showed a positive correlation with activation in medial BA 11. In contrast, Yang et al. (2015) asked participants to learn a list of words and make JOLs for only half of the words. Their results revealed that greater JOL accuracy was correlated with a smaller subsequent JOL effect (i.e., difference in activation between trials given high and low JOLs) in medial BA 11. One possible explanation for these conflicting results is that the different tasks used in different studies may affect the relationship between JOL accuracy and medial BA 11. Future studies should examine the effect of the task type on the relationship between JOL accuracy and medial BA 11.

Furthermore, our resting-state fMRI analysis showed that JOL accuracy was associated with a resting-state network that included the functional connectivity between the left mid-insula and different regions. These results are consistent with a previous study indicating that the mid-insula is functionally connected with various brain regions (Cauda et al., 2011). For example, Cauda et al. (2011) indicated that the anterior and posterior insula are involved in two different brain networks: the anterior insula is functionally connected to the frontal cortex, anterior cingulate cortex, parietal cortex and superior temporal gyrus, whereas the posterior insula is functionally connected to the sensorimotor cortex, the supplementary motor cortex and several regions in the temporal cortex and limbic system. In addition, Cauda et al. (2011) suggested that the mid-insula is a transitional area between the anterior and posterior insula and that the two networks overlap in the mid-insula. Thus, the mid-insula may be connected to regions in both networks. Furthermore, our results revealed that the functional connectivity found in this study could be divided into two subsets or factors (Factors 1 and 2; see **Table 2** and **Figure 4**). The functional connectivity within one factor was more closely correlated with each other than with the functional connectivity in the other factor, and the functional connectivity within a single factor may share similar functions (Jacobs et al., 2014). In addition, the two factors could independently predict individual differences in JOL accuracy, and a higher score of one factor was significantly correlated with a higher score of the other.

According to our factor analysis, Factor 1 contains the functional connectivity between the left mid-insula and various regions (including the left parahippocampal gyrus/fusiform gyrus/cerebellum, left SPL/Pcu, bilateral IPL/IPS and right frontal pole) and between the right frontal pole and the left SPL/Pcu. The parahippocampal gyrus is an important part of the medial temporal lobe and plays a key role in the memory encoding of various stimuli (Kirchhoff et al., 2000; Strange et al., 2002). The fusiform gyrus is related to the encoding of phonological or lexical features into episodic memory (Kirchhoff et al., 2000). In addition, the functional connectivity between the left mid-insula and left parahippocampal gyrus/fusiform gyrus is likely related to the posterior insula network, which may play an important role in sensorimotor integration (Cauda et al., 2011). It is possible that in the JOL process, the functional connectivity between the left mid-insula and the parahippocampal gyrus/fusiform gyrus may also be related to the integration of information about the encoding phase. In the JOL process, the left mid-insula may integrate the information about the phonological or lexical attributes of words (from the fusiform gyrus) and other information in the encoding process (from the parahippocampal gyrus), and higher efficacy of this information integration process may predict higher JOL accuracy. In addition, the parietal regions found in the present study (including the SPL, Pcu, IPL, and IPS) are closely associated with episodic memory (Wagner et al., 2005; Baird et al., 2013), and previous studies have revealed that regions in the dorsal parietal cortex, such as the SPL, Pcu, and IPS, are related to top-down attention in memory encoding (Cabeza et al., 2008; Uncapher et al., 2011). Moreover, the functional connectivity between the left mid-insula and left SPL/Pcu, bilateral IPL/IPS and right frontal pole may be related to the anterior insula network, which is associated with attention control (Cauda et al., 2011). The relationship between JOL accuracy and functional connectivity between these regions suggests that the control of our attention in the JOL process may support accurate JOLs. In addition, given the possible role of the mid-insula in the information integration process (Craig, 2011), another explanation for the role of the functional connectivity between the left mid-insula and these regions in the JOL process is that when JOLs are made, the left mid-insula may receive and integrate the information about attention control from these regions.

Moreover, the frontal pole has also been shown to be involved in the JOL process. For example, Ryals et al. (2016) found that transcranial magnetic stimulation (TMS) on the frontal pole could significantly increase JOL accuracy. The frontal pole is the apex of the rostro-caudal hierarchically organized prefrontal cortex (Badre, 2008; Badre and D’Esposito, 2009), and Ryals et al. (2016) suggest that the frontal pole may interact with other memory-processing regions in the JOL process and integrate information from these posterior regions. Thus, the functional connectivity between the right frontal pole and left SPL/Pcu may be associated with the integration of information in the JOL process. Taken together, the functional connectivity in Factor 1 may reflect the process of information integration, which is likely to support accurate JOLs.

In our factor analysis, Factor 2 contains the functional connectivity between the left SPL/Pcu and bilateral IPL/IPS. These regions mainly belong to the dorsal attention network (DAN) and the frontoparietal network (FPN) (Yeo et al., 2011). The DAN and FPN support the top-down control of attention and goal-directed processing (Dosenbach et al., 2007; Cabeza et al., 2008). Previous research has shown that selective attention toward the to-be-remembered stimuli can enhance the processing of the stimuli (Uncapher et al., 2011), which may have promoted JOL accuracy in the present study. Our results indicate that JOL accuracy may be related to the functional connectivity within the DAN and FPN, suggesting a possible role of selective attention in making accurate JOLs. Interestingly, the functional connectivity between the bilateral IPL/IPS in Factor 2 also had a factor loading higher than 0.3 on Factor 1, suggesting that this functional connectivity may be related to the integration of information in the JOL process. Future studies should further investigate the role of the bilateral IPL/IPS in the process of information integration when people make JOLs.

Our behavioral results indicated that individual differences in JOL accuracy did not correlate with recognition accuracy. In addition, we found that the GM volume and functional connectivity of the left mid-insula, which showed significant correlation with JOL accuracy, was not related to recognition accuracy, suggesting that the cognitive processes underlying JOL accuracy and memory performance might be distinct. Recent studies have obtained similar results with brain stimulation techniques (Chua and Ahmed, 2016; Ryals et al., 2016). For example, Ryals et al. (2016) found that TMS on the frontal pole could increase JOL accuracy but showed no effect on recognition performance. These results are also consistent with previous functional MRI studies showing that different brain regions are activated during memory process and JOL, indicating a dissociation between the two processes (Kao et al., 2005; Do Lam et al., 2012). According to the cue-utilization theory, participants do not base their JOLs on direct assessments of memory strength. Instead, their JOLs are based on various cues obtained in the encoding phase, and JOL accuracy depends on whether these cues can accurately predict memory performance (Koriat, 1997; Dunlosky and Metcalfe, 2009). Thus, JOL accuracy reflects the correspondence between JOL and memory performance (rather than memory performance itself), and the brain regions that are essential for JOL accuracy may not be involved in the memory process. Future studies should further disentangle the neural correlates of memory performance and JOL accuracy.

Our study has several limitations. First, the sample size (35 participants) in the present study was not large enough. Second, similar to all VBM and resting-state functional connectivity studies, our results are correlational in nature and do not imply any direct causality between individual differences in JOL accuracy and the GM volume of the left mid-insula or resting-state functional connectivity. Although individual differences in brain structure and resting-state activity are closely linked to differences in brain function (Kanai and Rees, 2011; Lee et al., 2013), more evidence is needed to further examine the results of this study and our explanation concerning the neural mechanisms related to individual differences in JOL accuracy. Third, our conclusions are not exhaustive, as they do not exclude the possibility that other regions may also be related to JOL accuracy without any difference in the GM volume or resting-state activity. Finally, we did not investigate whether individual differences in JOL accuracy are related to other cognitive functions. For example, a previous study suggested that executive function may be related to metamemory processes (Destan and Roebers, 2015). Future studies should use larger sample sizes and combine event-related fMRI, structural MRI and resting-state fMRI techniques to further examine the neural mechanisms concerning individual differences in JOL accuracy. In addition, future studies should also investigate the similarities and differences between the neural mechanisms of individual differences in JOL accuracy and other cognitive functions, such as executive function.

## Ethics Statement

This study was carried out in accordance with the recommendations of the Institutional Review Board of the State Key Laboratory of Cognitive Neuroscience and Learning, Beijing Normal University with written informed consent from all subjects. All subjects gave written informed consent in accordance with the Declaration of Helsinki.

## Author Contributions

XH and LL designed the experiment. XH, WC, JZ, NS, WW, and LL performed the experiment. XH, ZL, and LL analyzed the data. XH, ZL, CL, and LL wrote the manuscript.

## Conflict of Interest Statement

The authors declare that the research was conducted in the absence of any commercial or financial relationships that could be construed as a potential conflict of interest.

## Footnotes

1 http://dbm.neuro.uni-jena.de/vbm

2 http://www.fil.ion.ucl.ac.uk/spm/software/spm8/

3 http://www.alivelearn.net/xjview/

4 http://www.restfmri.net/forum/DPARSF
